# Exercise improves intestinal IgA production by T-dependent cell pathway in adults but not in aged mice

**DOI:** 10.3389/fendo.2023.1190547

**Published:** 2023-12-07

**Authors:** Angel Joel Hernández-Urbán, Maria Elisa Drago-Serrano, Andrea Cruz-Baquero, Ana Lilia García-Hernández, Ivonne Maciel Arciniega-Martínez, Judith Pacheco-Yépez, Fabiola Guzmán-Mejía, Marycarmen Godínez-Victoria

**Affiliations:** ^1^ Laboratorio de Citometria de Flujo e Investigación en Inmunología Clínica, Sección de Estudios de Posgrado e Investigación, Escuela Superior de Medicina, Instituto Politécnico Nacional, Mexico City, Mexico; ^2^ Departamento de Sistemas Biológicos, Universidad Autónoma Metropolitana, Unidad Xochimilco, Mexico City, Mexico; ^3^ Laboratorio de Inmunología en Enfermedades Infecciosas, Escuela Superior de Medicina, Instituto Politécnico Nacional, Mexico City, Mexico; ^4^ Programa Bacteriología y Laboratorio Clínico, Facultad de Ciencias de la Salud, Universidad Colegio Mayor de Cundinamarca, Bogotá, Colombia; ^5^ Laboratorio de Investigación Odontológica, Sección Osteoinmunologia e Inmunidad Oral, Facultad de Estudios Superiores Iztacala, Universidad Nacional Autónoma de Mexico, Mexico City, Mexico; ^6^ Laboratorio de Inmunonutrición, Sección de Estudios de Posgrado e Investigación, Escuela Superior de Medicina, Instituto Politécnico Nacional, Mexico City, Mexico

**Keywords:** aerobic moderate exercise, immunoglobulin A (IgA), T-dependent cell pathway, aging, gut associated lymphoid tissue (GALT), small intestine, Peyer´s patches

## Abstract

**Introduction:**

Hypermutated high-affinity immunoglobulin A (IgA), neutralizes toxins and drives the diversification of bacteria communities to maintain intestinal homeostasis although the mechanism underlies the impact of moderate aerobic exercise (MAE) on the IgA-generation via T-dependent (TD) is not fully know. Therefore, the aim of this study was to determine the effect of long-time MAE on the production of IgA through the TD pathway in Peyer´s patches of the small intestine from aged mice.

**Methods:**

MAE protocol consisted of twenty 3-month-old (young) BALB/c mice running in an endless band at 0° inclination and a speed of 10 m/h for 5 days a week and resting 2 days on the weekend until reaching 6-month-old (adulthood, n=10) or 24-month-old (aging, n=10). Groups of young, adult, or elderly mice were included as sedentary controls (n=10/per group). At 6 or 24 months old, all were sacrificed, and small intestine samples were dissected to prepare intestinal lavages for IgA quantitation by ELISA and to obtain suspensions from Peyer´s patches (PP) and lamina propria (LP) cells for analysis of T, B, and plasma cell subpopulations by flow cytometry and mRNA analysis expression by RT-qPCR of molecular factors related to differentiation of B cells to IgA+ plasma cells, class switch recombination, and IgA-synthesis. Statistical analysis was computed with two-way ANOVA (factor A=age, factor B=group) and p<0.05 was considered for statistically significant differences.

**Results:**

Compared to age-matched sedentary control, in exercised elderly mice, parameters were either increased (IgA concentration, IL-21, IL-10 and RDH mRNA expression), decreased (α-chain mRNA, B cells, mIgA+ B cells, mIgM+ B cells and IL-4 mRNA) or unchanged (PP mIgA+ plasmablasts and LP cyt-IgA+ plasma cells). Regarding the exercised adult mice, they showed an up-modulation of IgA-concentration, mRNA expression IL-21, IL-10, and RDH and cells (PP B and T cells, mIgM+ plasmablasts and LP cyt-IgA+plasma cells).

**Conclusion:**

Our findings suggest that MAE restored the IgA production in adult mice via the TD cell pathway but does not in aged mice. Other studies are necessary to know in more detail the impact of long-time MAE on the TD pathway to produce IgA in aging.

## Introduction

Immunoglobulin A (IgA) is a key player in mucosal immune regulation through several mechanisms: 1) immune exclusion via interacting with environmental antigens (e.g., bacteria, viruses, and toxins); 2) anti-inflammation by sampling intestinal antigens to induce Th2 or regulatory T cell-biased mucosal immune responses; 3) homeostasis of commensals by enhancing the cross talk between the probiotic bacteria and the intestinal mucosa (1). Thus, the lack of IgA in the intestine is associated with various intestinal diseases, such as necrotizing enterocolitis and gastrointestinal mucositis (2). Targets to increase the secretion of intestinal IgA are promising and directed at mitigating the pathogenesis of diseases (1).

One route of IgA generation encompasses the T dependent (TD) pathway that takes place in the interfollicular region of the Peyer’s patches (PP) regarded the main compartment of gut-associated lymphoid tissue (GALT). TD pathway generates high affinity and hypermutated IgA and entails antigen sampling of M cells overlying the luminal surface of PP. M cells continuously sample and transport luminal antigens to the underlying PP subepithelial dome enriched in dendritic cells (DC) that act as potent antigen-presenting cells that, after capturing and processing antigens, undergo maturation. Mature DCs express on the cell surface complexes such as peptide-major histocompatibility class II antigens (MHC-II) and CD80/CD86 and then, migrate to the interfollicular zone to present antigens to T cells expressing on the membrane the T cell receptor (TCR) and CD28. Immunological synapsis among cognate ligand-receptor ligations of peptide-MHC-II -TCR and CD80/CD86-CD28, drive signal pathways resulting in the generation of T helper (Th) effector cells. The cells migrate to PP germinal zone to interact with B cells expressing membrane IgM (mIgM)+ B cells via cognate ligand-receptor interactions TCR-MHC-II and CD40L-CD40 that provide signals for the activation in an environment rich in transforming growth factor-β (TGF-β), to induce the increase in the expression of AID and TSLP and carry out the class switch recombination (CSR) of IgM→IgA, where the expression of CXCR5 (dependent on Bcl-6), PD-1 and the secretion of IL-21 in the Lfh, as well as a microenvironment rich in IL-4, IL-10, TGF-β and AR, are necessary for this CSR, differentiation and clonal proliferation of antigen-specific B plasmablasts expressing membrane IgA (mIgA+ plasmablast cells); and ultimately production of IgA by lamina propria (LP) IgA+ B cells (3). Hypermutation and IgA class-switching of B cells are determined by enzymes such as activation-induced cytidine deaminase (AID) whose expression is enhanced by retinal dehydrogenase (RDH), TGF-β and interleukin-21 (IL-21) secreted by T follicular Th cells (Tfh). Moreover, Tfh secrete cytokines such as IL-5, IL-6, and IL-10 that elicit the clonal proliferation and maturation of mIgA+ B cell plasmablasts. Once the class-switch and differentiation of mIgM+ B cell to mIgA+ B cells are carried out, mIgA+ B plasmablasts migrate through the bloodstream or lymphatic to LP where they are differentiated into plasma cells secreting soluble IgA (3). At LP, IgA acts as a ligand of the polymeric immunoglobulin receptor (pIgR), expressed at the basolateral membrane of epithelial cell monolayer, that carries out the vesicle-mediated IgA transport toward the luminal milieu a process known as transcytosis; at the apical surface, the extracellular ligand binding portion of pIgR is cleaved by proteolytic enzymes and released as secretory component (SC) in free form or as part of secretory IgA (SIgA) in the lumen.

Currently, exercise has been promoted as a strategy to strengthen immunity against diseases associated with aging caused, in part, by the functional imbalance of the immune system, a process known as immune senescence (4, 5). The contributions regarding the effects of exercise on the intestinal immune response made by our working group show that moderate exercise promotes the increase of intestinal levels of IgA in the duodenum and ileum of the small intestine of adult mice (6, 7). This increase was accompanied by an increase in mRNA expression of mediators involved in the generation (TGF-β, IL-4, and IL-10) and in the transport of IgA through pIgR (IL-4, TNF-α, and IFN-γ (6, 7). Other trials showed that moderate exercise plus a final session of intense acute exercise favors the increase of IgA however reduces the transcriptional expression of most mediators that promote its generation and transport in young adult mice (6 months of age) (8).

Other studies in experimental animal models and human trials have reported that exercise increases salivary levels of IgA in the elderly associated with protection against upper respiratory tract infections (9–11). The impact of moderate exercise on IgA production in the gut in the elderly population is not fully known. Although such findings indicate that exercise is an important modulator of the adaptive immune system, the cellular and molecular mechanisms underlying its mode of action remain unclear. This study aims, for the first time, to assess the effect of performing a moderate aerobic exercise from young age to aging on the production of intestinal IgA through the T cell-dependent pathway in BALB/c mice. This could promote and strengthen intestinal protection and homeostasis and reduce the development of several diseases in elderly people.

## Materials and methods

### Animals

Forty 6-week-old BALB/c mice were kept in groups of 5 mice per cage, with light-dark cycles of 12:12 h (7AM/7PM), little noise, and minimal manipulation at the time of cleaning of cages. Mice were fed with Laboratory Rodent Diet 5001 (LabDiet, Saint Louis MO, USA) and water *ad libitum*, and kept on a natural dark-light cycle. Manipulation and the exercise protocol were conducted between 8 AM and 11 AM to avoid the influence of circadian cycles of ACTH and corticosterone (12). The procedures carried out on the animals complied with the requirements that determine the standard: NOM-062-ZOO-1999 “Technical specifications for the production, care, and use of laboratory animals” SAGARPA; and the “Guide for the Care and Use of Laboratory Animals”. This protocol was approved by the Institutional Committee for the Care and Use of Laboratory Animals of the Higher School of Medicine of the National Polytechnic Institute, Mexico with register key: ESMCICUAL_04/08-11-2020.

### Moderate exercise protocol

Before starting the exercise protocol, mice underwent a period of adaptation during weeks 9 and 10 of life. The adaptation period consisted of a 10-minute session of exercise in endless band at 0° of inclination with increments of 5 min every other day until reaching 30 min/day. After that, mice ran in an endless band at 0° of inclination and a speed of 10 m/h. During the entire study, mice ran 5 days per week and rested for 2 days until 6 or 24 months old (13).

### Experimental design

Mice were adapted to their environment for 2 weeks before starting the exercise protocol in week 9 of life. For that, animals were divided into 2 groups: 1) sedentary control mice and 2) exercised mice (n=20 per group). From each group, 10 mice were sacrificed at 6 months (adult mice) and the other 10 at 24 months (elderly mice). The other group of 3-month-old (young mice, n=10), after the adaptation period, was included as basal control of aging.

### Samples

At 6 or 24 months of age, the sedentary and exercise mice were sacrificed by administering a lethal dose of sodium pentobarbital at 150 mg/kg body weight, by intraperitoneal via. Blood was obtained by intracardiac puncture to separate the serum to determine the cortisol concentration. After that, the small intestine was dissected, and 5 ml of PBS containing protease inhibitor cocktail (cat. no. 11836153001, Complete mini Roche Mannheim Germany cat. Roche^®^) was passed through intraluminal space to obtain the intestinal flush to determine the IgA concentration by ELISA. Afterward, the PP were cut and disaggregated to flow cytometric and RT-qPCR analysis. Once the PP were removed, lamina propria were obtained using two different density gradients, as has been previously described (14), and cells were analyzed by flow cytometry or stored at -70°C to await analysis by RT-qPCR.

### Detection of the IgA protein by ELISA

Total IgA antibody concentration was determined in fluids from the small intestine by using the ELISA assay, according to a previously reported method (15, 16).

### Lymphoid sub-populations in Peyer´s patches and lamina propria by a cytofluorometric assay

The percentage of the following cell populations was determined: T cells [CD3+ cells], CD4+ T cells, Tfh cells (CD3+/CD4+/CXCR5+ cells), CD40L expression on CD4 T cells, naïve B cells (IgM+/IgD+/B220+/CD19+), mIgA + B cells (IgA+/B220+/CD19+), mIgA+ plasmablast cells (IgA+/CD138+/B220-) present in PP and cytoplasmic-IgA (cyt-IgA) plasma cells (IgA+/CD138+) in LP of the small intestine using the flow cytometry technique. The staining of the lymphocytes was carried out using the next anti-mouse antibodies obtained from Becton Dickinson Pharmigen, San Diego, CA: PECy7-CD3 (cat. 552774), APC-H7-CD4 (cat. 560181), PECy7-CD19 (cat. 561739), CD45R/B220 (cat. 553089), APC-IgD (Cat. 560868), PerCP-Cy5.5-IgM (cat. 550881) and FITC-IgA (cat. 559354). Antibodies PerCP-Cy5.5-CXCR5 (cat. 560528) and PE-CD154 (cat. 561719) were purchased from BD Biosciences, San Diego, CA, USA. The protocol provided by the manufacturer was used for surface staining. After staining plasma cells from LP, cells were permeabilized and fixed with cytoper/cytofix kit (cat. 554722 BD) for cyt-IgA determination. Samples were acquired and analyzed in a BD FACSAria Fusion flow cytometer using FACSDiva software. Ten thousand events in the lymphocyte region in the SSC vs. FSC dot-plot for T and B cells, and 20,000 events for plasma cells were analyzed.

### Relative expression of mRNA assay by RT-qPCR

The relative expression of the α-chain of IgA and AID, RDH, TGF-β1, IL-4, IL-6, IL-10, and IL-21 mRNA was determined in PP. Total RNA extraction was done using the protocol described for the fabricant of Trizol® reagent (cat. 15596018, Invitrogen). Reverse transcription was performed using the Maxima First cDNA Synthesis Kit for RT–qPCR (cat. No. K1642, Thermo Scientific, Lithuania) at 37°C for 50 min.

Quantitative PCR was subsequently performed by duplicate, using a Maxima Probe/ROX qPCR Master mix Kit (cat. K0261, Thermo Scientific™, Lithuania) in a LightCycler ® Nano Instrument (Roche Diagnostics GmbH, Rotkreuz Switzerland) at an initial denaturing step (at 95°C for 10 min), followed by 45 cycles of amplification (at 95°C for 10 s, 60°C for 35 s, and 72°C for 1 s) and one cycle of cooling (40°C for 30 s). Specific oligonucleotide primers were originally generated by using the online assay design software (ProbeFinder: http://www.universal-probelibrary.com) and the primer sequences for AID, RDH, and IL-21 are shown in **Table 1**. The primer sequences of α-chain, IL-10, IL-4, IL-6, and TGF-β have been previously published (8). mRNA expression levels were calculated using the 2^ΔΔCt method and normalized to the relative expression of GAPDH mRNA (17).

**Table 1:** Forward and reverse primers for real-time PCR assays designed according to the ensemble transcript ID of the Universal ProbeLibrary.

Gen		Sequence 5´→3´	ID*
**AICDA**	L	TCC TGC TCA CTG GAC TTC G	NM_009645.2
	R	GCG TAG GAA CAA CAA TTC CAC	
**IL-21**	L	TCC ATG TTG TGT CCG GGT A	NM_001291041.1
	R	TCT GTG GGA ACG AGA GCC TA	
**GAPDH**	L	AAG AGG GAT GCT GCC CTT AC	NM_001289746.1
	R	CCA TTT TGT CTA CGG GAC GA	

*Result from ProbeFinder version 2.53 for Mouse. AICDA, activation-induced cytidine deaminase; IL, interleukine; GAPDH, glyceraldehyde-3-phosphate dehydrogenase; (L), left; (R), right.

### Statistical analysis

At the end of the study (at 24 months old), the survival was 60% in the control group and 80% in the exercised group. For that, data are presented as the mean and standard deviation (SD) of 6–8 animals per group. For the comparison between groups in the different measurement times, two-way ANOVA was used considering as factors the group (sedentary or exercise) and the age of the mice (young, adult, and elderly mice), followed by a Turkey method *post hoc* test. For all tests, p< 0.05 was considered significant. All data were analyzed using SigmaPlot for Windows version 11.1 (Systat Software Inc. San Jose CA, USA).

## Results

The results represent the effect of long-term MAE performed from youth (3 months old) to adulthood (6 months old) or elderly (24 months old). All data were compared to results obtained in young mice which are considered the baseline parameters. The effect of age was analyzed by comparing elderly versus young and adult groups; the effect of exercise was seen by comparing exercised vs sedentary groups.

### Moderate aerobic exercise increased intestinal IgA concentration in adult and senile mice

**Figure 1:**
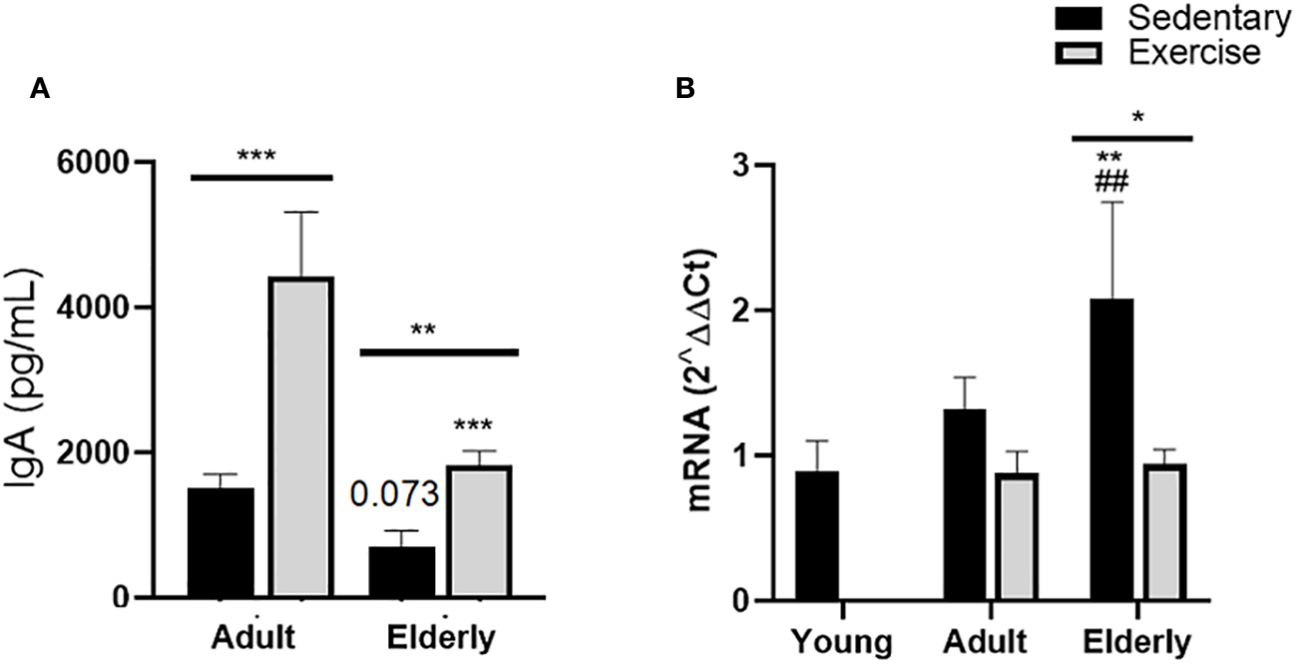
Immunoglobulin A expression in small intestine of sedentary or exercised mice from young age to senile age. Data represent the mean and standard deviation of **(A)** total IgA concentration (pg/mL) in intestinal liquid; **(B)** relative units of α-chain mRNA expression in Peyer´s patches. (##) versus sedentary young mice; (*) versus respective adult control; line on the bars, versus respective sedentary control. ##p<0.01, *p<0.05, **p<0.01 and ***p<0.001.

Analysis between the paired matched age groups indicated that IgA concentration was greater in exercised versus sedentary groups (adult p<0.001, elderly p<0.01). Comparisons within both exercised groups evidenced that IgA concentration was lower in the elderly versus adult groups (p<0.001, **Figure 1A**). Comparisons between sedentary groups showed that IgA concentration was not significantly different in the elderly versus the adult group (p=0.073, **Figure 1A**).

Comparative analysis in elderly mice of α-chain mRNA expression was found greater in sedentary than exercised mice (p<0.05); within sedentary groups, α-chain mRNA expression was higher in the elderly sedentary group than young and adult mice (p<0.01, **Figure 1B**).

### Moderate aerobic exercise decreased mIgA+ and mIgM+ B cell % in Peyer´s patches from senile mice

**Figure 2:**
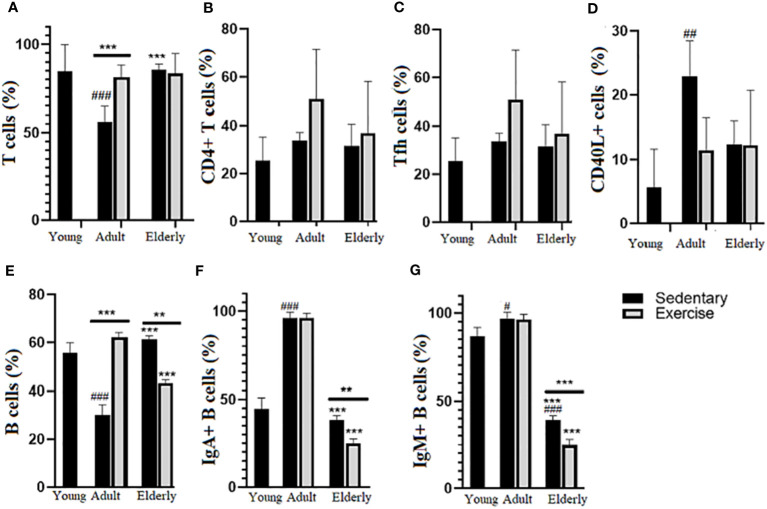
Percentage (%) of lymphoid subpopulations in Peyer´s patches from small intestine of sedentary and exercised mice from young to senile age. Data represent the mean and standard deviation of **(A)** total T cells; **(B)** CD4+ T cells; **(C)** follicular CD4+ T cells (Tfh); **(D)** CD40L+ CD4+ T cells; **(E)** total B cells; **(F)** mIgA+ B cells; **(G)** mIgM+. (#) versus sedentary young mice; (*) versus respective adult control; line on the bars, versus respective sedentary control. ##p<0.01, ###p<0.001, **p<0.01 and ***p<0.001. mIgA, membrane IgA+ cells; mIgM, membrane IgM+ cells.

Analysis of T cell % in adult groups indicated it was greater in exercised than sedentary group (p<0.001, **Figure 2A**), in elderly mice significant differences between exercise versus sedentary were unseen. Comparisons among the sedentary groups showed that T cell % was higher in elderly than adult mice and lower in adult than young mice (p<0.001, **Figure 2A**), no significant changes in CD4+T cell % (**Figure 2B**) and Tfh cells % (**Figure 2C**) were seen in all groups. Regarding CD40L+ T cells % in sedentary groups, it was found higher in adult than young mice (p<0.01, **Figure 2D**).

Comparisons within the adult groups evidenced that B cell % was greater in exercised than sedentary mice while in elderly mice it was lower in exercised than sedentary groups (adult p<0.001; elderly p<0.01, **Figure 2E**); within the exercised groups, B cell % was lower in elderly than adult mice (p<0.001) and within the sedentary groups B cell % was higher in elderly than adult mice or lower in adult than young mice (p<0.001 **Figure 2E**). Within elderly groups, mIgA+B cell % (p<0.01) and mIgM+ B cell % (p<0.001) were lower in exercised than sedentary groups; within the exercised groups, mIgA+ B cell % and mIgM+ B cell % were lower in elderly than adult mice (p<0.001) and among sedentary groups mIgA+ B cell % and IgM+ B cell % in elderly were lower than adult and young mice (p<0.001) and higher in adult than young mice (mIgA+ B cell % p<0.001, mIgM+ B cell % p<0.05) (**Figures 2F, G**).

### Moderate aerobic exercise did not significantly change the plasma cell % in elderly mice

**Figure 3:**
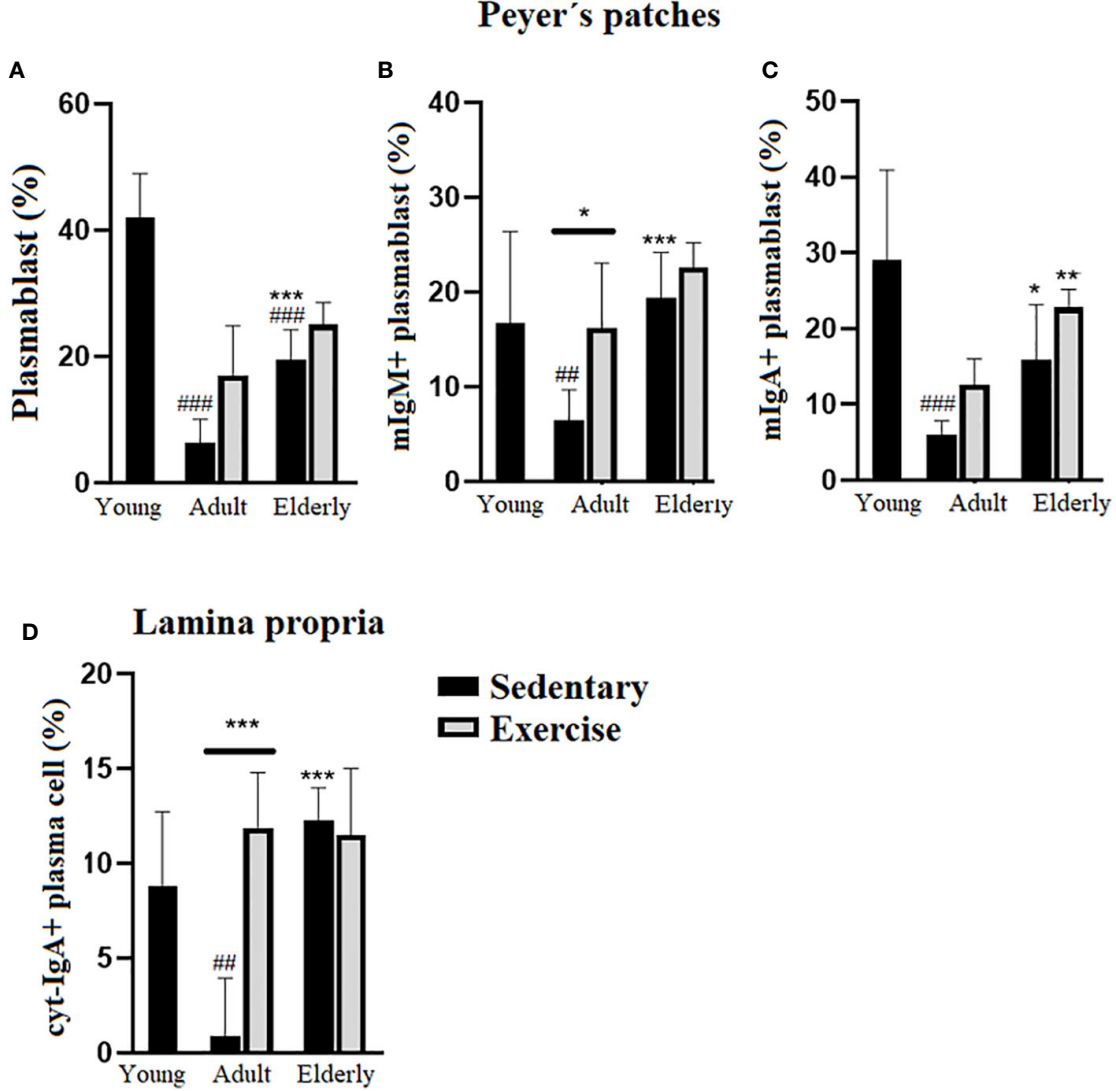
Percentage (%) of plasmablasts in Peyer´s patches and plasma cells in lamina propria of small intestine from sedentary or exercised mice at different ages. Data represent the mean and standard deviation of **(A)** total plasmablasts; **(B)** mIgM+ plasmablasts and **(C)** mIgA+ plasmablasts in Peyer´s patches and, **(D)** cyt-IgA+ plasma cells in lamina propria. (#) versus sedentary young mice; (*) versus respective adult control; line on the bars, versus respective sedentary control. ##p<0.01, ###p<0.001, *p<0.05, **p<0.01 and ***p<0.001. mIgA+ plasmablast, membrane IgA+ plasmablasts cells; mIgM, membrane IgM+ plasmablasts cells; cyt-IgA+ plasma cells, cytoplasmic IgA+ plasma cells.

Within sedentary groups, PP plasmablast cell % was higher in elderly than young and adult mice or lower in adult than young mice (p<0.001 **Figure 3A**). Within adult mice, PP mIgM+ plasmablast cell % was higher in exercised than sedentary mice (p<0.05), within the sedentary groups PP mIgM+ plasmablast cell % was greater in elderly than adult group (p<0.001) or lower in adult than young mice (p<0.01 **Figure 3B**). Analysis within the exercised groups evidenced that PP mIgA+ plasmablast cell % was greater in elderly than adult mice (p<0.01), within the sedentary groups PP mIgA+ plasmablast cell % was higher in elderly than adult (p<0.05) or lower in adult than young mice (p<0.001 **Figure 3C**). Comparisons in adult mice indicated that LP cyt-IgA+ plasma cell % was greater in exercised than sedentary mice (p<0.001); within the sedentary groups LP cyt-IgA+ plasma cell % was higher in elderly than adult mice (p<0.001) or lower in adult than young mice (p<0.01 **Figure 3D**).

### Moderate aerobic exercise modulated differential factors associated with IgA-generation in adult and elderly mice

**Figure 4:**
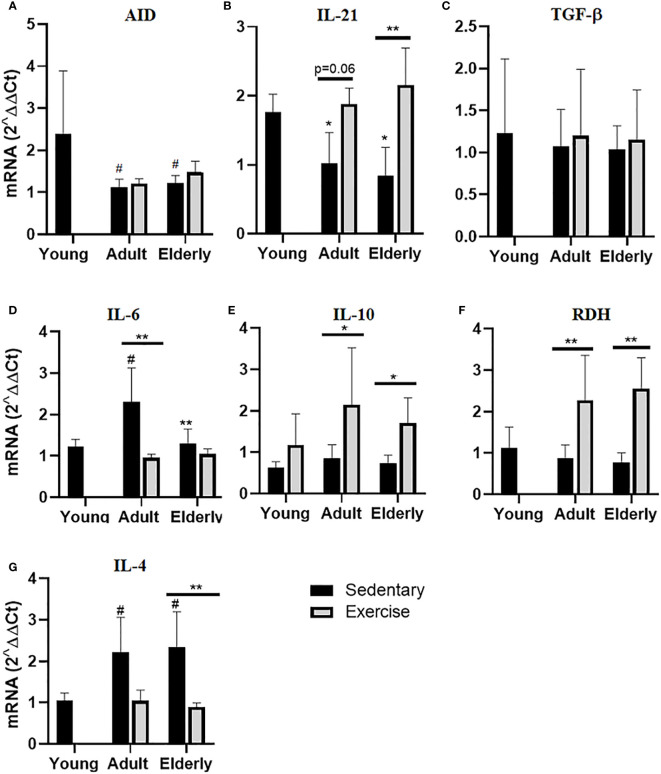
Gene expression of factors related to production of hypermutated IgA in Peyer´s patches of small intestine from sedentary or exercise mice at different ages. Data represent the mean and standard deviation of **(A)** activation-induced cytidine deaminase (AID), **(B)** interleukin (IL)-21, **(C)** transforming growth factor-β (TGF-β), **(D)** IL-6, **(E)** IL-10, **(F)** retinol dehydrogenase (RDH) and **(G)** IL-4 mRNA expression. (*) versus respective adult control; line on the bars, versus respective sedentary control. #p<0.05, *p<0.05 and **p<0.01.

Within sedentary groups, AID mRNA expression was lower in elderly and adult versus young mice (p<0.05 **Figure 4A**). In elderly groups IL-21 mRNA expression was higher in exercised than sedentary mice (p<0.01) and within sedentary groups, IL-21 mRNA expression was lower in elderly and adult versus young mice (p<0.05 **Figure 4B**). No significant changes in TGF-β were unseen in all groups (**Figure 4C**). In adult mice, IL-6 mRNA expression was greater in sedentary than exercised mice (p<0.01) while in sedentary mice IL-6 mRNA expression was lower in elderly than adult (p<0.01) or higher in adult than young mice (p<0.05 **Figure 4D**). Analysis of IL-10 mRNA expression within paired matched age groups indicated greater IL-10 mRNA expression in exercised than sedentary mice (p<0.05 **Figure 4E**). Within the paired matched age groups, RDH mRNA expression was found greater in exercised than sedentary groups (p<0.01), and in sedentary groups RDH mRNA expression was lower in adult and elderly versus young mice (p<0.05 **Figure 4F**). Lastly in elderly mice, IL-4 mRNA expression was lower in exercised than sedentary mice (p<0.01) and within sedentary groups IL-4 mRNA expression was higher in adult and elderly than young mice (p<0.05 **Figure 4G**).

## Discussion

It is now recognized that physical activity has a substantial impact on components of intestinal immunity, microbiota, and IgA production in aging (18, 19). Although at present the results about the pathways involved in the production of IgA in the small intestine of senile mice are minimal. In this context, the most relevant contributions of this research are: 1) the effect of aging on the production of IgA via T-dependent cells and 2) the effects of long-term moderate aerobic exercise on the regulation of alterations caused by aging on the T-dependent cell production of intestinal IgA.

This study was focused on assessing for the first time the effect of moderate exercise on the IgA T-dependent response in senile mice. In comparison to a sedentary counterpart, in elderly exercised mice, we observed an effect of the exercise on the up-modulation of IgA concentration along with the increase of mRNA expression of IgA-associated humoral factors (IL-21, IL-10, and RDH). The impact of aging in the increased IgA concentration was previously documented in aged mice (20). In our study, exercise does not counteract the effect of aging on the decline of α-chain mRNA expression and B cell populations (total plasmablast, mIgM+, and mIgA+ plasmablast) in Peyer´s patches. Moreover, the failure of exercise on immune restoration in aging was also evidenced by a decrease in IgA concentration, B cells, mIgA+, and mIgM+ plasmablasts regarding exercise conditions in adult age. The effect of aging on the downmodulation of intestinal IgA and/or IgM antibodies and IgA+ and/or IgM+ secreting cells at PP has been reported before in experimental models of aged mice (21–23). Apparent discordances among increase of IgA concentration, decrease of IgA secreting cells as well as α-chain mRNA expression reflect the aging outcome on intestinal immunity dysregulation, and they may include: i) alternative sources of IgA production like isolated lymphoid follicles as described in aged mice (20, 24) ii) alternative routes of IgA production such as T-independent pathway (TI) in aged nu/nu mice (24). iii) a redistribution of cells among lymphoid tissues which must be mainly due to the release of stress hormones such as catecholamines and glucocorticoids which has been reported in exercised mice (25). iv) permeability increased by aging as documented in human trials of aging (26). Furthermore, it is known that exercise can impact mucosa immunity through the activation of the hypothalamus-hypophysis-adrenal axis resulting in the release of stress hormones like cortisol. In this regard, a significant effect of moderate exercise on the decrease of cortisol levels in aging may be beneficial for bone structure as documented previously by our research team (13) Cortisol is considered an aging marker and high levels of cortisol are directly proportional to higher aging and cellular damage (27–29). The Last result lets us consider exercise as a good strategy to decrease the impact of aging on overall health. In brief, our results suggested that under conditions of moderate exercise, aging does not promote the IgA-generation via-dependent pathway.

Compared to adult sedentary control in PP, the exercise in adult mice increased the IgA concentration along with the increase of T cells, B cells, PP IgM+ plasmablast, and LP IgA+plasma cells; aforementioned were along with the increased IL-21, IL-10, and RDH mRNA levels. Our findings matched with the outcome of moderate exercise in adult mice on the increase of IgA-associated parameters (6, 7). Data suggest that exercise re-established the TD cell pathway to produce IgA in adult mice at levels found in young mice moreover, exercise decreased IL-4 mRNA expression thus the generation of IgA plasma cell precursors may be favored. It is known that IL-4 is regarded as a negative regulator of IgA plasma cell precursors (30). A significant effect of moderate exercise on the increase of serum cortisol was documented previously in adult mice (13). The role of cortisol on the IgA-generation via TH2 subpopulation was documented in nasal-associated lymphoid tissue from mice that underwent acute stress that induced an increase of plasma corticosterone and promoted the Th2 and Treg response (characterized by an increase of IL4, IL-5, and IL-10). This microenvironment favors the IgA biosynthesis by CD138+IgA+ plasma cells, which is a process mediated by glucocorticoids (31). Briefly, our data suggested in adulthood moderate exercise promotes the IgA-generation via-dependent pathway. Moderate exercise in adulthood may be beneficial to strengthen mucosal immunity via the TD pathway.

Among the sedentary groups, the outcome of aging regarding adult age on the decrease (IgA concentration (apparent), mIgA+/mIgM+ plasmablasts) or increase (T cells, B plasmablasts, PP mIgA+/mIgM+ cells and LP cit IgA plasma cells) suggested age-associated dysregulation on these parameters as described before [decrease (21–23) and increase (20)].

Data indicated that in comparison to young mice, aging had a significative outcome on the decrease of mIgM+ B cells, plasmablast cells, AID, IL-21, and RDH mRNA expression. This evidence suggests that aging blunted the TD pathway for IgA generation. Furthermore, by comparison with young mice, aged mice showed no differences among most parameters analyzed (IgA concentration, IL-6, IL-10, TFG-β, LP plasma cells, and T cells) although some others were increased (α-chain, IL-4 RNA expression). In brief, all these aforementioned findings suggested that aging favored IgA production via the TI pathway under sedentary conditions. Our findings somewhat agree with published data, where it has been observed that aging increases the repertoire of diversity by a dual process: on the one hand dependent on the microbiota, T cells, and the transcription factor RORt and on the other in a form independent of the somatic mutation in PP which is probably contributing to the generation of IgA via TI pathway (32). Furthermore, the expression of chemokines such as CCL25 in the small intestine is associated with a reduction in IgA-producing plasma cells and IgA concentration in the intestinal lumen, favoring the deterioration of intestinal immunity in aging (33). In this contribution protein analysis by Western blot to evaluate α-chain and ELISA for determining IgA antibodies to fecal microbiota was not done, in spite of these limitations, the study showed that moderate exercise favored the TD pathway in adult and not in aged mice.

## Conclusions

Moderate aerobic exercise performed constantly from young to senile age reduced the impact of aging on intestinal immunity by maintaining stable production of IgA.

The results of this research suggest that moderate exercise did not have effects on the TD pathway for IgA generation because the cellular and expression of factors were blunted under conditions of aging.

Although there are some differences in relation to the immune systems of mice and humans, these results could serve as an experimental basis for establishing preventive health programs where the practice of moderate aerobic exercise is considered and especially encouraged from the early stages of life. Particularly in the case of elderly people, the current COVID-19 pandemic emergency has strongly demonstrated that older individuals are more fragile when challenged by an unknown pathogen, fostering the need to understand which mechanisms differentiate the aged immune system from that of a young individual (34).

## Data availability statement

The original contributions presented in the study are included in the article/supplementary material. Further inquiries can be directed to the corresponding author.

## Ethics statement

The animal study was approved by Institutional Committee for the Care and Use of Laboratory Animals of the Higher School of Medicine of the National Polytechnic Institute, Mexico with register key: ESMCICUAL_04/08-11-2020. The study was conducted in accordance with the local legislation and institutional requirements.

## Author contributions

AJH-U, ALG-H, IMA-M, AC-B, and JP-Y contributed to the acquisition and analysis of data. FG-M contributed to drafting the work and revised it critically for important intellectual content. MED-S and MG-V made a substantial contribution to the conception or design of the work; interpretation of data for the work, drafting the work, and revising it critically for important intellectual content; providing approval for publication of the content; agreeing to be accountable for all aspects of the work in ensuring that questions related to the accuracy or integrity of any part of the work are appropriately investigated and resolved. All authors contributed to the article and approved the submitted version.
